# Research challenges and opportunities in the Caribbean area: first bibliometric study in the French West Indies, from 1989 to 2018

**DOI:** 10.26633/RPSP.2021.159

**Published:** 2021-12-22

**Authors:** Cédric Contaret, Raymond Cesaire, Jacqueline Deloumeaux, Rémi Neviere, Dabor Resiere, André Cabie, Emmanuelle Sylvestre, Clarisse Joachim, Moustapha Drame

**Affiliations:** 1 Centre Hospitalier Universitaire de Martinique, Fort de France Martinique France Centre Hospitalier Universitaire de Martinique, Fort de France, Martinique, France.; 2 Université des Antilles, Pointe-à-Pitre Guadeloupe France Université des Antilles, Pointe-à-Pitre, Guadeloupe, France.; 3 Centre Hospitalier Universitaire de la Guadeloupe, Pointe-à-Pitre Guadeloupe France Centre Hospitalier Universitaire de la Guadeloupe, Pointe-à-Pitre, Guadeloupe, France.

**Keywords:** Collaboration indicator, bibliometrics, Caribbean region, Guadeloupe, Martinique, Indicador de colaboración, bibliometría, región del Caribe, Guadalupe, Martinica, Indicador de colaboração, bibliometria, região do Caribe, Guadalupe, Martinica

## Abstract

**Objective.:**

To analyze, describe, and quantify the collaborations and scientific output of the two university teaching hospitals of Martinique and Guadeloupe, at the regional, national, and international level.

**Methods.:**

A bibliometrics analysis was performed from the international databases Web of Science and PubMed, for the period from 1989 to 2018, inclusive (30 years). Three types of bibliometric indicators were used, namely quantitative indicators, performance indicators, and organization-specific indicators. Affiliations of the first and last authors were identified from PubMed.

**Results.:**

Between 1989 and 2018, a total of 1 522 indexed articles were published with at least one author affiliated to either the University Hospital of Martinique (n = 827) or the University Hospital of Guadeloupe (n = 685). The majority of articles were in category Q1 (35.8% for Martinique and 35.2% for Guadeloupe). In Martinique, over the last 30 years, the three main research areas have been clinical neurology, ophthalmology, and surgery, together representing 28.7% of all research areas, with the highest number of articles published in the field of clinical neurology (n = 81). In the University Hospital of Guadeloupe, the area of hematology was largely represented, with 79 articles published. For both hospitals, the first and last authors of the articles published were mainly from mainland France.

**Conclusions.:**

This quantitative analysis shows the development of medical and scientific research in Martinique and Guadeloupe over the last three decades, as well as the extent of their collaborative partnerships at the national and international levels.

## RESUMEN

**Objetivo.:**

Analizar, describir y cuantificar las colaboraciones y la producción científica de los dos hospitales docentes universitarios de Martinica y Guadalupe, al nivel regional, nacional e internacional.

**Métodos.:**

Se llevó a cabo un análisis de bibliometría de las bases de datos internacionales Web of Science y PubMed, en el período de 1989 al 2018, inclusive (30 años). Se emplearon tres tipos de indicadores bibliométricos, a saber: indicadores cuantitativos, indicadores de rendimiento e indicadores específicos de organización. Se establecieron las afiliaciones de los primeros y últimos autores de PubMed.

**Resultados.:**

Entre 1989 y el 2018, se publicaron en total 1 522 artículos indizados con al menos un autor afiliado al Hospital Universitario de Martinica (n = 827) o bien al Hospital Universitario de Guadalupe (n = 685). La mayoría de los artículos estaban en el cuartil 1 (35,8 % en Martinica y 35,2 % en Guadalupe). En Martinica, en los últimos 30 años las tres principales áreas de investigación han sido la neurología clínica, la oftalmología y la cirugía, que juntas representan 28,7 % de todas las áreas de investigación, con el número más alto de artículos publicado en el campo de la neurología clínica (n = 81). En el Hospital Universitario de Guadalupe, el ámbito de la hematología estaba ampliamente representado, con 79 artículos publicados. En ambos hospitales, los primeros y los últimos autores de los artículos publicados eran principalmente de Francia continental.

**Conclusiones.:**

Este análisis cuantitativo demuestra el desarrollo de la investigación médica y científica en Martinica y Guadalupe en los treinta últimos años, así como el alcance de sus asociaciones colaborativas a los niveles nacional e internacional.

## RESUMO

**Objetivo.:**

Analisar, descrever e quantificar as colaborações e a produção científica dos dois hospitais universitários de Martinica e Guadalupe, no âmbito regional, nacional e internacional.

**Métodos.:**

Foi realizada uma análise bibliométrica das bases de dados internacionais Web of Science e PubMed, no período de 1989 a 2018, inclusive (30 anos). Três tipos de indicadores bibliométricos foram utilizados: quantitativos, de desempenho e específicos da organização. Afiliações dos primeiros e últimos autores foram identificadas na base PubMed.

**Resultados.:**

Entre 1989 e 2018, um total de 1 522 artigos indexados foram publicados com pelo menos um autor afiliado ao Hospital Universitário de Martinica (n = 827) ou ao Hospital Universitário de Guadalupe (n = 685). A maioria dos artigos estava na categoria Q1 (35,8% para Martinica e 35,2% para Guadalupe). Na Martinica, nos últimos 30 anos, as três principais áreas de pesquisa foram neurologia clínica, oftalmologia e cirurgia, representando, juntas, 28,7% de todas as áreas de pesquisa, com o maior número de artigos publicados no campo da neurologia clínica (n = 81). No Hospital Universitário de Guadalupe, a área de hematologia foi amplamente representada, com 79 artigos publicados. Para ambos os hospitais, os primeiros e os últimos autores dos artigos publicados eram principalmente da França continental.

**Conclusões.:**

Esta análise quantitativa demonstra o desenvolvimento da pesquisa médica e científica na Martinica e em Guadalupe nas três últimas décadas, bem como a extensão de suas parcerias colaborativas no âmbito nacional e internacional.

Bibliometrics are mathematical methods used in various scientific domains to measure scientific output based on the number of publications, the prestige of the journal, and the number of citations of the research after its publication (1, 2). Several recent bibliometric studies have evaluated scientific repercussions or temporal trends in different areas of scientific and medical research (3). Other studies have focused on the impact of publication output of one or several institutions (4, 5) or at a national level (6). To the best of our knowledge, only a few bibliometric studies have been performed in the Caribbean or the French West Indies (7, 8).

For more than 30 years, national plans have been implemented in France to develop research within French hospitals, including in the regions of Guadeloupe and Martinique (French West Indies, FWI). These two regions are not geographically located in Europe, but rather, are situated in the Caribbean. University teaching hospitals were set up in 1985 in Guadeloupe and in 1986 in Martinique. The implementation of successive programs to boost attractiveness and to support emerging research teams has culminated in the existence of several research units that are certified by the French Ministry for Research and Higher Education (9), which are a rich breeding ground for research in these two regions. Indeed, the Caribbean zone presents several specific epidemiological features that represent major public health issues. Due to their geographical position, the FWI are exposed to severe climatic conditions that promote emerging vector-borne diseases (Chikungunya, dengue, Zika), as well as being exposed to meteorological phenomena such as earthquakes and hurricanes. Hurricanes have become more frequent and increasingly severe in recent years. There is therefore a compelling need for close cooperation between Caribbean countries to face these threats. Furthermore, the burden of chronic diseases (obesity, diabetes, cancer) is growing, and at the origin of substantial morbidity (10). Populating ageing is another major preoccupation for both islands, and is a priority topic for research, with the planned constitution of population cohorts in the coming years (11, 12).

Since 2008, in both Guadeloupe and Martinique University Hospitals, new organizational structures for the promotion of research have been put in place, such as the Delegation for Clinical Research and Innovation (Délégations de la Recherche Clinique et de l’Innovation, DRCI), Clinical Investigation Centers, and centers for biological resources. These new structures have been a major driving force behind the local development of research activities (13). The DRCI’s purpose is to provide support on a daily basis for all those involved in developing research projects, from the conception of the idea and writing of the protocol, through submission to calls for projects at regional, interregional and European level, and right up to publication of the final results, with staff members specialized in maximizing output and impact (14). The aim is to be capable of competing for large-scale financing programs, such as the French government’s national program for hospital research (Programme Hospitalier de Recherche Clinique, PHRC), or European Union funds allocated to research, training, and development.

Taken together, these specificities of the FWI underscore the importance of medical and scientific cooperation between Caribbean countries and the need for a concerted effort between the ministries of health and research in neighboring countries, which should be facilitated by Martinique and Guadeloupe’s recent joining of the Organisation of Eastern Caribbean States (OECS), in 2015 and 2019, respectively (15).

To date, to the best of our knowledge, no bibliometric study has investigated scientific collaboration between the various academic institutions of the FWI and their scientific output. Given the specific epidemiological characteristics of Martinique and Guadeloupe, it would be interesting to assess the development of research in these two regions. The aim of this study was therefore to perform a descriptive analysis by quantifying the collaborations and scientific output of the two university teaching hospitals of Martinique and Guadeloupe, at the regional, national, and international level.

## MATERIALS AND METHODS

We performed a bibliometrics analysis from the international databases Web of Science and PubMed, for the period from 1989 to 2018 inclusive (30 years). All original research articles, reviews, letters, editorials, and comments were selected; other publication types were excluded.

To search for publications involving the University Hospital of Martinique and/or the University Hospital of Guadeloupe, we used InCites, an analytical tool developed and marketed by Clarivate Analytics (16) that aggregates summary measures of scientific output and citations at the level of countries, organizations, and specific disciplines.^1^

An organization enhanced feature available in the Web of Science allows the user to search by an organization name that has been unified, to yield comprehensive and specific search results. The terms CHU de Martinique and CHU de Guadeloupe were searched in the “organization” section in the InCites database. Web of Sciences used more than 215 different search terms in search engines for these terms. All Medline-indexed publications were extracted in Excel format. Publications were extracted in XML (Extensible Markup Language) format from PubMed (17).

Three types of bibliometric indicators were used, namely quantitative indicators, performance indicators, and organization-specific indicators. The quantitative indicator was the number of publications per five-year period, to measure scientific output in each hospital by type of publication. Performance indicators were: (1) the quality of publications, as measured by the average journal impact factor; and (2) the average number of citations of the article per five-year period. The “Top 1%” and “Top 10%” indicators systematically provided by InCites were also used to identify the most highly cited articles at the international level. The Journal Citation Reports were used to rank journal by quartiles of impact factor, with journals in Q1 (i.e., the top 25%) corresponding to the most widely cited journals in the field.

Organization-specific indicators, also known as structural indicators, were used to measure connections between publications, authors, and research fields. To estimate the structural indicators, the full set of data for each article identified was extracted from PubMed. Affiliations of the first and last authors were identified from PubMed. Each affiliation thus identified was classed by country. Martinique, Guadeloupe, as well as French Guiana, Réunion island, Mayotte, and French Polynesia were all identified with codes distinct from that of metropolitan France, to make it possible to distinguish them and compare with mainland France.

Between 2014 and 2018, we recorded the affiliations of all authors (from the first to the last author) who collaborated on an article with the University Hospital of either Martinique or Guadeloupe. For authors with several affiliations, the hospital affiliation was retained.

Results are presented in table format for quantitative and qualitative indicators, and in graphical format for organization-specific indicators. Data were extracted in XML format from PubMed and analyzed using SAS version 9.4 (SAS Institute Inc., Cary, NC).

## RESULTS

Between 1989 and 2018, a total of 1 522 indexed articles were published with at least one author affiliated to either the University Hospital of Martinique (n = 827) or the University Hospital of Guadeloupe (n = 685). For both hospitals, there was a steady increase in the number of articles published over the years, reaching a peak in the most recent period (i.e., 2014–2018, with 348 articles for the University Hospital of Martinique and 292 for Guadeloupe).

Quantitative indicators are presented in Table 1. Almost 80% of publications were original research articles in both hospitals. For both sites, the language used in each publication was predominantly English (67% for Martinique and 78% for Guadeloupe), the other language being French. For Martinique, the proportion of articles in journals ranked in the top two quartiles increased steadily, from 21.4% and 10.7%, respectively, in the first five-year period, to 40.0% and 25.1% in the last five-year period. The majority of articles for both hospitals were in category Q1 (35.8% for Martinique and 35.2% for Guadeloupe).

**FIGURE 1.**
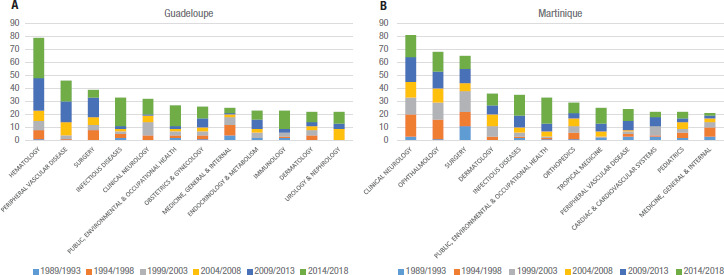
Distribution of articles published by the university hospitals of Guadeloupe (panel A) and Martinique (panel B) by research area, 1989 to 2018

***Source*:** Prepared by the authors from the study results, based on published data.

Figure 1 presents the number of articles published by research area over the study period for the two hospitals, with authors in first or last position. In Martinique, over the last 30 years, the three main research areas have been clinical neurology, ophthalmology, and surgery, together representing 28.7% of all research areas, with the highest number of articles published in the field of clinical neurology (n = 81). In the University Hospital of Guadeloupe, the area of hematology was largely represented, with 79 articles published. There was a substantial increase in the number of articles published in the most recent five-year period, in the area of infectious diseases for Guadeloupe, and in the field of public, environmental, and occupational health for Martinique, with the number of articles multiplied by 2 and 1.5 respectively between 2014 and 2018. Only the 12 most widely represented research areas are shown in Figure 1.

**FIGURE 2.**
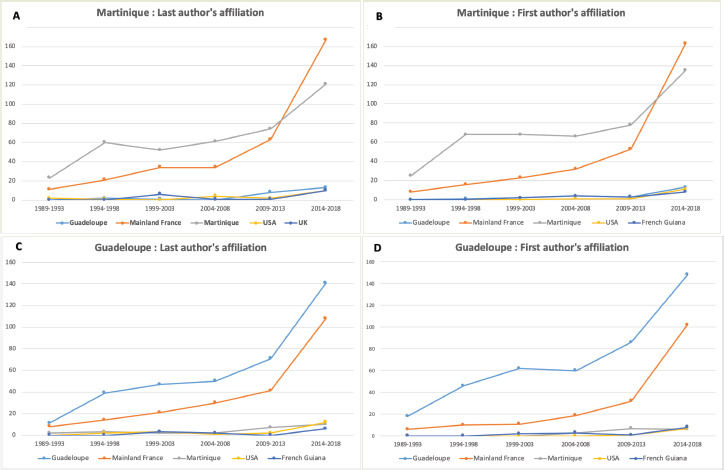
Evolution of the number of articles according to the ranks of authors in Martinique (A and B) and in Guadeloupe (C and D)

***Note*:** UK, United Kingdom; USA, United States of America.

***Source*:** Prepared by authors from the study results, based on published data.

Figure 2 illustrates the five most frequently represented countries among the affiliations of the first authors, and among the affiliations of the last authors, for the articles attributed to the University Hospital of Guadeloupe and for those attributed to the University Hospital of Martinique over the last 30 years. For both hospitals, the first and last authors of the articles published were mainly from mainland France, and from either Martinique or Guadeloupe university hospital. In articles attributed to Guadeloupe, the first author was actually from Guadeloupe in 65.8% (420 authors) and the last author in 56.3% (n = 359). In Martinique, the distribution was similar, with 55.8% of first authors (n = 440) and 49.6% of last authors (n = 391) from Martinique. During the most recent five-year period of the study, there was a net increase in the number of first and last authors from mainland France in articles from both hospitals: from 2009–2013 to 2014–2018, there was a 2.86-fold increase in the number of authors from mainland France in publications from Martinique, and a 2.91-fold increase in publications from Guadeloupe, versus 1.68-fold and 1.85-fold increases for authors originating from Martinique and Guadeloupe, respectively.

Detailed analysis of the bibliographic metrics is shown in Table 2. For both hospitals, the average number of authors per article was lower when an author from that hospital was first or last author. The average number of authors per article was highest when the first or last author was from mainland France.

## DISCUSSION

Over the last 30 years, the scientific output of the university hospitals of Martinique and Guadeloupe has increased steadily. The increase in the number and quality of publications over the last three decades was particularly marked in the last five-year period of the study (2014–2018). This could be due to the implementation of local policies promoting research, or to the increase in the number of physicians with academic teaching positions in both regions, thanks to the creation of the Faculty of Medicine in the FWI in 2001. Furthermore, advances in medicine and technological innovation are also factors that should be taken into account in the booming research activity at a national scale. With the help of various programs implemented by the Ministry of Higher Education and Research to restructure research activities in France, successive strategic plans have led to the emergence of centers of excellence, in which research and public health issues are the foundation for their work in advancing the state of knowledge. Previous studies have also reported a strong increase in the number of scientific articles published in recent years in large institutions (4, 5).

Analysis of the position of each author signing the publications is an indicator of the level of involvement of the various research teams in the work. This simple indicator reveals that when authors from Martinique and/or Guadeloupe are in first or last position, then the average number of authors from both sites is relatively low. Collaborations with authors from other countries could help to increase the number of citations per article, thereby markedly increasing the visibility of the publications (18, 19).

In the period from 2014 to 2018, the proportion of authors from the Caribbean islands was low, at around 4% in collaboration with the University Hospital of Martinique, and 9% with the University Hospital of Guadeloupe. Yet, public health problems that are common to all the Caribbean islands are a strong impetus for scientific cooperation that feature in the public health strategic plans laid down by the Caribbean Public Health Agency (CARPHA) (20) and regional health authorities (21).

Creating research consortia that comprise researchers from different research institutes could help them to mutually contribute to advancing scientific knowledge specific to their geographical area. Several cooperative initiatives in oncology have been reported in recent publications, leading to the set-up of a consortium bringing together Cuba, Martinique, and Puerto Rico for the surveillance of cancer through population-based registries (22). In terms of the environment, the recent sargassum seaweed invasion along the coasts of the Caribbean islands (23) could prompt international multidisciplinary collaboration (24). The emergence of arboviral diseases in Caribbean countries (25–27) was also an example showing the importance for these countries to collaborate and pool their research efforts in this public health issue. The dynamic of scientific collaboration within the Caribbean is a real challenge for both sites of the FWI. Indeed, the language barrier with its Caribbean neighbors, the sociocultural differences, and health care and demographic contexts are heterogeneous across the Caribbean, and in fact, are potentially brakes to this collaboration (28). It is noteworthy that cooperation between the FWI and its Caribbean neighbors has already been initiated with the recent integration of Martinique and Guadeloupe into the Organisation of Eastern Caribbean States (OECS), in 2015 and 2019, respectively (29).

**TABLE 1.** Description of the bibliometrics indicators of scientific output for the University Hospital of Martinique and the University Hospital of Guadeloupe, 1989 to 2018

	Martinique							Guadeloupe						
	Total	1989–1993	1994–1998	1999–2003	2004–2008	2009–2013	2014–2018	Total	1989–1993	1994–1998	1999–2003	2004–2008	2009–2013	2014–2018
**Number of articles**	**837**	38	86	100	108	157	348	**685**	24	67	77	88	137	292
**Research article**	**651**	27	65	86	88	129	256	**548**	15	52	55	80	115	231
**Letter**	**89**	5	14	11	8	13	38	**84**	6	8	15	5	12	38
**Review**	**44**	1	0	2	8	6	27	**22**	0	0	4	2	5	11
**Editorial**	**42**	1	0	1	4	9	27	**23**	0	2	3	1	5	12
**Comment**	**11**	4	7	0	0	0	0	**8**	3	5	0	0	0	0
**Funding projects**	**152 (18%)**	0	0	0	0	41 (26%)	111 (32%)	**154 (22%)**	0	0	0	5(5%)	35(25%)	114 (39%)
**Impact factor (Median; IT)**	**2.56; 3.19**	1.96; 2.45	1.16; 1.51	1.54; 2.58	2.44; 3.10	2.56; 2.84	2.77; 3.10	**2.42; 3.06**	2.55; 1.94	1.79; 2.67	2.17; 3.40	2.30; 4.46	2.19; 2.96	2.76; 3.20
**Time cited (Median; IT)**	**4.00:12.00**	3.00;15.00	5.00:9.00	7.50;18.00	8.00;25.00	7.00;17.00	2.00;6.00	**6.00;13.00**	3.00;14.00	8.00;14.00	8.00;17.00	10.00;18.00	6.00;14.00	3.00;8.00
**Q1 (%)**	**35.82**		21.43	24.44	35.00	37.32	40.00	**35.23**		34.78	37.10	31.08	26.61	40.41
**Q2 (%)**	**21.19**		10.71	17.78	20.00	17.61	25.16	**20.83**		17.39	14.52	24.32	22.58	20.82
**Q3 (%)**	**12.24**		32.14	15.56	8.00	10.56	11.61	**19.32**		21.74	22.58	17.57	19.35	18.78
**Q4 (%)**	**30.75**		35.72	42.26	37.00	34.51	23.23	**24.62**		26.09	25.81	27.03	31.45	20.00
**Top 10% (%)**	**10.56**	5.26	6.02	8.00	7.48	10.83	13.79	**10.06**	15.00	3.39	6.85	6.25	9.09	13.48
**Top 1% (%)**	**2.04**	0	0	4	0	1.91	2.87	**0.93**	0	1.69	0	0	0	1.77

**TABLE 2.** Analysis of the affiliations of authors in all articles published from 2014 to 2018

		Martinique (number of articles: 348)					Guadeloupe (number of articles: 292)				
		**Affiliations**					**Affiliations**				
**Total number of authors**		Mainland France	Martinique	Guadeloupe	USA	French Guiana	Mainland France	Guadeloupe	Martinique	USA	French Guiana
	n (%)	2 159 (54.7)	1 020 (25.84)	124 (3.14)	115 (2.9)	91 (2.3)	1 496 (50.3)	967 (32.5)	122 (4.1)	66 (2.2)	64 (2.15)
	Mean ± SD	23 ± 15	10 ± 7	19 ± 15	17 ± 6	15 ± 6	25 ± 20	10 ± 7	15 ± 10	12 ± 8	13 ± 7
**First authors**		**Affiliations**					**Affiliations**				
		Mainland France	Martinique	USA	Guadeloupe	French Guiana	Guadeloupe	Mainland France	Martinique	USA	French Guiana
	n (%)	163 (47.0)	132 (38.0)	10 (2.9)	9 (2.6)	8 (2.3)	141 (48.3)	108 (37.0)	10 (3.4)	12 (3.6)	6 (2.05)
	Mean ± SD	14 ± 11	7 ± 5	19 ± 9	10 ± 7	13 ± 4	7 ± 4	14 ± 12	14 ± 11	9 ± 6	11 ± 3
**Last authors**		**Affiliations**					**Affiliations**				
		Mainland France	Martinique	Guadeloupe	UK	USA	Guadeloupe	Mainland France	Martinique	French Guiana	USA
	n (%)	168 (48.4)	121 (34.9)	12 (3.5)	11 (3.2)	9 (2.6)	148 (50.7)	102 (34.9)	6 (2.0)	8 (2.7)	7 (2.4)
	%	48.41	34.87	3.46	3.17	2.59	50.68	34.93	2.05	2.74	2.40
	Mean ± SD	14 ± 11	7 ± 5	8 ± 4	10 ± 11	16 ± 6	7 ± 4	15 ± 13	10 ± 5	10 ± 3	10 ± 7

***Notes***: USA, United States of America; SD, standard deviation.

Total number of authors: total number of authors from each of the five most frequently represented countries in the affiliations of all authors from all publications from the university hospitals of Martinique (left panel, n = 348) and Guadeloupe (right panel, n = 292).

First authors: Among the first authors of all publications from Martinique (n = 348) or Guadeloupe (n = 292), the five most frequently represented countries are listed, with the number of authors from each, and the average number of authors on the publication with the first author from the country cited.

Last authors: Same analysis as for first authors but considering only the last authors on the publications.

***Source*:** Prepared by authors from the study results, based on published data.

In Guadeloupe, drepanocytosis is the historical research topic, bolstered by the presence of both a national reference center and a National Institute of Health and Medical Research (INSERM) research unit in Guadeloupe. Collaborations initiated across the Caribbean through the formation of CAREST, the Caribbean Network of Researchers on Sickle Cell Disease and Thalassemia, and with certain African countries, are starting to pay dividends in terms of scientific publications. The topic of cardiovascular and metabolic diseases, both prevalent problems in Guadeloupe and Martinique (e.g., type 2 diabetes, hypertension, chronic renal insufficiency), was long epitomized by a hospital-based research team that made a significant contribution to the visibility of this theme through scientific publications in reputed journals. Over the last decade, the problem of environmental pollution from pesticides has attracted an INSERM unit to set up in Guadeloupe, focusing on the topic of health, environment, and work. Collaborations with local teams have also been prolific in terms of number of publications. Finally, more recently, the major upsurge in emerging infectious diseases, notably epidemics of Chikungunya and Zika viruses, have propelled the FWI to center stage in terms of research and boosted the number of scientific publications with international collaborations. This wide spectrum of research areas in these two regions needs to be supported by strong research institutions, working in synergy, to meet the challenges of the island context and relatively small populations. This corroborated one of the difficulties of the FWI to obtain funding specific to their local public health problems (12). French or European financial funds could be directed toward research themes arising from public health problems that strongly affect European countries. Indeed, the publication of articles in underdeveloped research fields, despite the high prevalence of certain diseases, would lead to an increase in the number of publications by local university researchers, thus contributing to the scientific influence of West Indian research. The fact that the majority of scientific production is by authors from mainland France can also be explained by the small number of practitioner-researchers in the two sites (30).

In view of the size of the population, with an estimated 770 000 inhabitants as of 1 January 2016 in Martinique and Guadeloupe together, the level of technical equipment also has to take account of the critical mass of patients necessary to maintain certain medical specialty services, or to render further investment and development viable. Specialties that are offered in these two regions therefore develop in synchrony, working in complementarity as regards the health care opportunities on offer in each island (specialists, number of positions, and technical equipment). As a result, which disciplines will produce scientific publications is often a question of the geographical location of the experts in that particular field. This explains the predominance of certain research themes across the two regions.

Among the factors that need to be taken into account, it is necessary to consider the amount of time that health care professionals have available to engage in research. Indeed, in such ultraperipheral regions as Martinique and Guadeloupe, the attractiveness of the health care environment for professionals is lower (31, 32), and there is high staff turnover and geographical isolation; factors that all have negative repercussions on research efforts within the hospitals in these regions. The unfavorable state of the medical demographics is at the root of the severe difficulties experienced in implementing and sustaining research projects. Indeed, it is not just a question of initiating projects on research areas of interest, but they must also be brought to a successful conclusion and achieve appropriate publication and visibility.

The main partner for both Guadeloupe and Martinique university hospitals over the last 30 years has been mainland France. The participation of both islands in publications reporting multicenter projects led by teams from metropolitan France is very frequent, not to say the majority of publications for the University Hospital of Martinique in the last five years. This type of collaborative partnership ensures better visibility and provides an opportunity to gain experience of innovative, large-scale projects, which is an asset in terms of partnership potential. The hospitals of Guadeloupe and Martinique thus work mainly with a partner situated around 8 000 km away and work considerably less with their geographically closer Caribbean neighbors, who share similar demographic characteristics.

Regarding the types of research projects carried out in each of the two hospitals studied, access to funding relies on the availability of permanent research teams, as their durability over time is a key factor in successfully completing and publishing research. The recent developments in the university organization in the FWI led to the emergence of research programs headed by national teams, and which allowed for the inclusion of patients from the West Indies. Progressively, research teams within the Guadeloupe and Martinique hospitals were thus able to develop their own research topics, with their hospitals starting to act as sponsor for new studies. This increase in the number of studies sponsored by the hospitals of Martinique and Guadeloupe subsequently led to the initiation of multicenter projects at national and international level, in a positive knock-on effect.

A potential limitation of this study is that it does not take account of possible explanatory factors such as human relations, which can underpin the initiation of cooperative projects. It would have been interesting to conduct a survey among the health care professionals in both institutions, investigating the barriers and facilitating factors for scientific research between the two regions, in order to gain a broader understanding of the factors influencing publication strategies in both hospitals.

In terms of future perspectives, the dissemination of our findings among the university hospital research teams will be an essential step in understanding how to work toward more collaborative partnerships in the future. Among the possibilities for cooperation, participating in European partnership programs such as the European Regional Development Fund (ERDF) or European Territorial Cooperation (ETC) (e-health CARES 2014–2020) gives improved visibility to our islands’ ability to lead operational projects on topics such as the health care pathway, access to technological innovation, or e-health. The research areas targeted by these programs are issues that are common to the whole region, or that aim to exploit the specificities of the region, and public health is one of these. It therefore behooves the research teams in both Martinique and Guadeloupe to demonstrate unequivocally their ability to rise to the strategic challenge of research and innovation.

## Conclusion

To the best of our knowledge, this is the first study to perform a bibliometric analysis of the scientific output of the FWI over a period of 30 years. This quantitative analysis showed the development of medical and scientific research in Martinique and Guadeloupe over the last three decades, as well as the extent of their collaborative partnerships at national and international level. Our findings shed light on some shortfalls, namely the need to implement more joint cooperative projects in order to join forces in facing up to the growing burden of chronic and emerging diseases, whose prevalence is high in the Caribbean. In the long term, integrating bibliometric indicators into routine practice would make it possible to closely monitor research and innovation strategies across these two regions. Future investigations in this area could focus on analysis of clinical trials reported at the national and international level, to assess the level of involvement of the FWI and help to better define objectives for future collaborations and publications.

## Disclaimer.

Authors hold sole responsibility for the views expressed in the manuscript, which may not necessarily reflect the opinion or policy of the *Revista Panamericana de Salud Pública/Pan American Journal of Public Health* and/or those of the Pan American Health Organization.

## Footnotes

1 Search strategy and keywords used are available from the authors upon request.
